# Mesenteric Lipoma-Induced Small Bowel Obstruction in a Pediatric Patient: A Rare Case of Midgut Volvulus

**DOI:** 10.7759/cureus.80058

**Published:** 2025-03-04

**Authors:** Rafael A Guzman, Bryan A Wallace, Feras Othman, Charissa Lake, Juan Calisto

**Affiliations:** 1 General Surgery, St. George's University School of Medicine, True Blue, GRD; 2 General Surgery, University of Miami, Coral Gables, USA; 3 Pediatric Surgery, Nicklaus Children's Hospital, Miami, USA

**Keywords:** lipoma, mesenteric lipoma, pediatric case, pediatric lipoma, small bowel obstruction, volvulus of midgut

## Abstract

Mesenteric lipomas are rare benign tumors composed of adipocytes, representing a small subset of intestinal lipomas. Although typically asymptomatic, large lipomas can cause significant complications, such as small bowel obstruction (SBO), particularly when they induce bowel volvulus. We present a case of a 10-year-old female with no notable past medical history who developed SBO due to a mesenteric lipoma causing volvulus. This case highlights a rare condition with a unique clinical presentation that is not commonly seen in the literature. Imaging studies, including ultrasound, abdominal X-ray, and contrast-enhanced CT, identified a fat-dense mass in the right lower quadrant (RLQ) that led to small bowel rotation and obstruction. Surgical resection confirmed the diagnosis of mesenteric lipoma. This case emphasizes how significant complications can develop, even in the setting of benign tumors. Given the potential for serious complications, such as midgut volvulus and SBO, further research into the clinical presentation, diagnostic strategies, and management of mesenteric lipomas in pediatric populations is essential to improve patient outcomes and guide future clinical practice.

## Introduction

Lipomas are benign tumors composed of adipocytes that can manifest anywhere there are fat cells in the body, including the mesentery of the bowel [1]. Mesenteric lipomas are rare as there are only a handful of cases published in the English literature, especially in the pediatric population. The pathophysiology of a lipoma is unclear, but links have been made to genetic anomalies and trauma [1]. Symptoms associated with mesenteric lipomas can include abdominal pain, constipation, and vomiting [2-3]. Symptoms are highly dependent on mass size and location as mass effect can cause a variety of presentations. Mesenteric lipomas can be diagnosed using imaging such as computed tomography (CT), magnetic resonance imaging (MRI), and ultrasound (US) [4]. X-ray can be used to diagnose associated conditions such as bowel obstructions. Surgical resection is the treatment of choice, and a definitive diagnosis can be obtained through pathological analysis of specimens [2-4]. The purpose of this study is to showcase a rare case of mesenteric lipoma presenting with small bowel obstruction (SBO) due to volvulus in a pediatric patient.

## Case presentation

**Figure 1:**
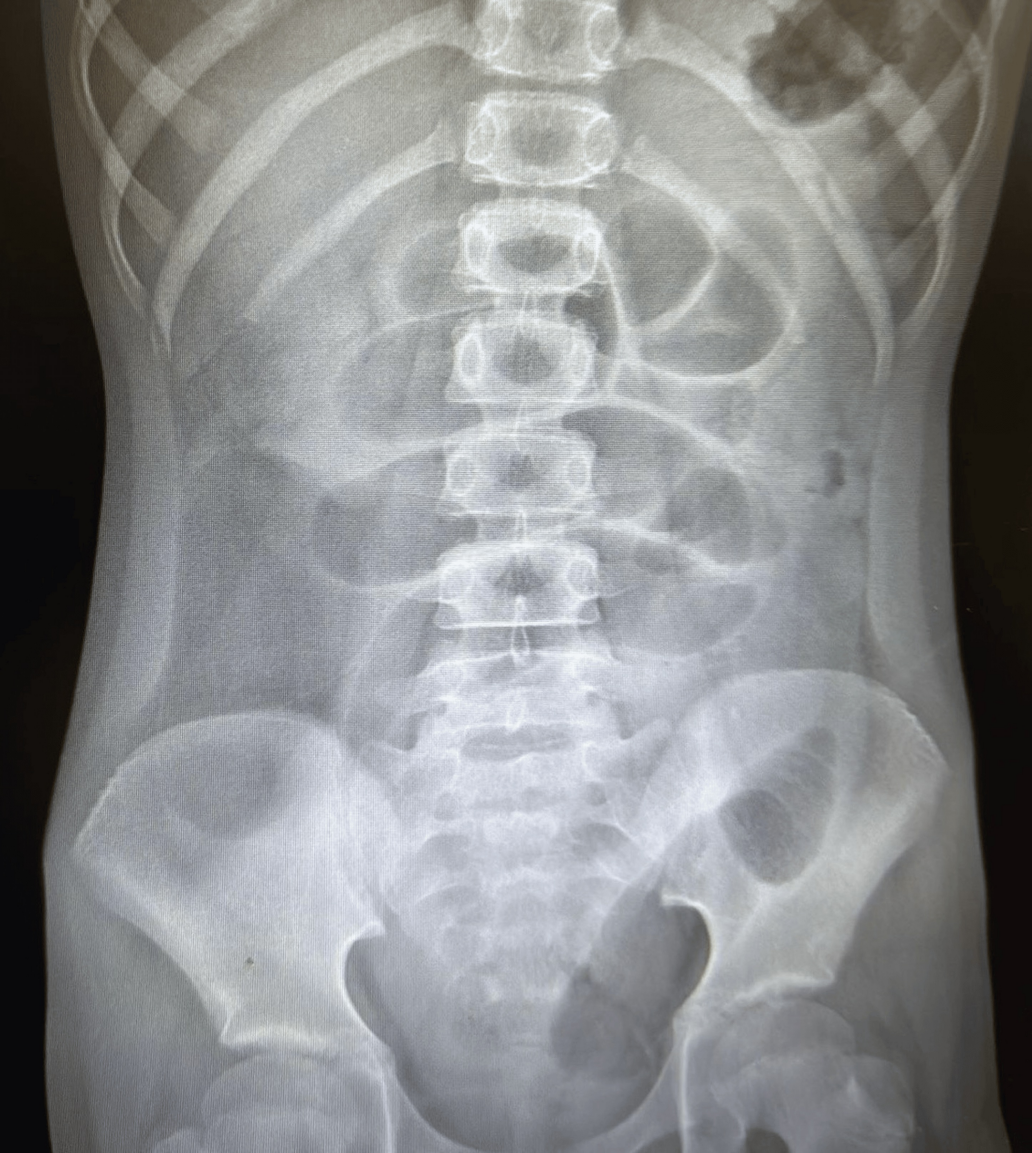
Abdominal X-ray showing multiple air-fluid levels.

A 10-year-old female patient with no notable past medical history (PMH) presented to the emergency department with her father for evaluation and management of generalized abdominal pain and vomiting. Per the father, the patient had a one-day history of abdominal pain associated with nine episodes of non-billious, non-bloody vomiting. He also stated that the patient had not had a bowel movement in three days, which is atypical of her. On physical examination, the abdomen was mildly distended and tender to palpation at the epigastric and periumbilical area. No signs of peritonitis. Ultrasound showed a mass-like structure in the right lower quadrant (RLQ) measuring 11 x 6 x 8 cm with no associated features and a normal-looking appendix. Abdominal X-ray showed multiple air fluid levels (Figure 1). 

CT abdomen/pelvis showed a fat-dense mass in the RLQ measuring 10.1 x 5.7 x 6.8 cm, evidence of mass effect on the right colon, dilated fluid-filled loops, and small amounts of free fluid in pelvis and RLQ. Vital signs showed tachycardia. Labs showed direct hyperbilirubinemia and neutrophilia (Table 1).

**Table 1:** Laboratory results.

Labs	Patient	Normal
Direct bilirubin	0.7 mg/dL	>0.3 mg/dL
White blood cells	10.4×10³/µL	4.5-11.0×10³/µL
Neutrophil differential	85.9%	25.0-70.0%
Hemoglobin	15.2 g/dL	12.0-15.5 g/dL
C-reactive protein	0.5 mg/dL	<1.0 mg/dL

**Figure 2:**
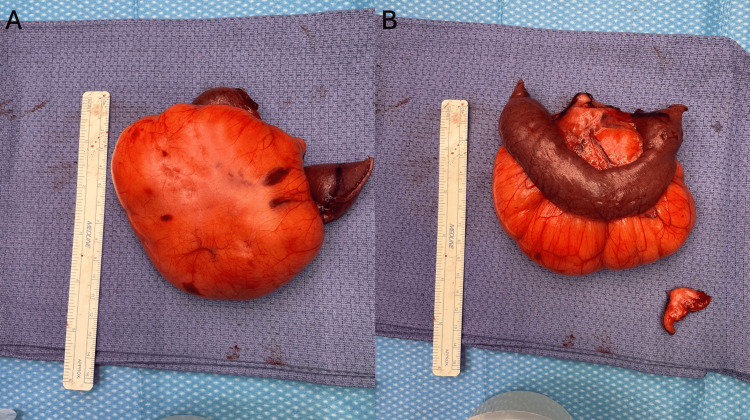
Post-resection lipoma and portion of small bowel. (A) and (B) show different views of the same resected mass and portion of small bowel.

The patient was taken for a diagnostic laparoscopy, which revealed the mass within the mesentery of the jejunum twisted around the root of the mesentery. The operation was converted to exploratory laparotomy where the mass, a portion of the small bowel, mesentery, and one enlarged lymph node were removed and sent for pathological analysis (Figure 2). 

Pathology showed mature adipocytes surrounded by thin fibrous capsules and resected node with benign sinus histiocytes, confirming the diagnosis of lipoma. The postoperative course was unremarkable.

## Discussion

We report a rare case of a mesenteric lipoma causing a volvulus in a 10-year-old patient. In the pediatric population, 64-80% of cases of a primary midgut volvulus present within their first month of life, with 90% occurring in infants within their first year [5]. A midgut volvulus is usually triggered by a congenital anomaly where there is an aberrant malrotation on the axis of the mesentery during the development of the gastrointestinal system [5-6]. However, a midgut volvulus can occur at any age. For adults, a volvulus commonly develops secondarily due to postoperative adhesions in the abdomen [7]. What makes this case even less common is the cause as to why this young patient developed a midgut volvulus with no significant past medical history to help aid in a diagnosis.

This patient presented with the core symptoms of an SBO: abdominal pain, vomiting, and constipation. While the patient was stabilized, an abdominal ultrasound and abdominal X-ray were taken, which were suggestive of an SBO. However, a computed tomography scan (CT scan) of the abdomen and pelvis was able to confirm a lipomatous mass as the cause of this patient's SBO. There are various underlying pathologies that may result in or predispose a patient to an SBO, the majority are caused by postoperative intestinal adhesions, which account for over 50% of SBOs [8]. However, intestinal lipomas are considered rare, with an incidence of 0.035-4.4% [9]. Intestinal lipomas are benign adipose growths that slowly develop within the wall of the small bowel or the mesentery and are usually asymptomatic and found incidentally on abdominal imaging [9]. As with our patient, this lipoma grew unnoticed to a substantial size within the mesentery, causing the small bowel to rotate on its axis, leading to a symptomatic bowel obstruction.

As with most symptomatic bowel obstructions, emergent assessment and management are warranted to avoid further complications in the bowel. If not corrected, the twisting of the bowel on its axis can cut off the flow of blood, allowing ischemia and necrosis of the small bowel to set in [6]. While the approximate size can be determined through abdominal imaging, the extent of bowel rotation and weight of the lipoma can only fully be assessed via a diagnostic surgical resection of the mass. Like so, during the laparoscopic removal of this patient's mesenteric lipoma, the surgical team noted the extent of the mass involvement of surrounding abdominal structures, and conversion to an open exploratory laparotomy was indicated.

As mentioned previously, lipomas are mesenchymal soft tumors of adipocytes. There are few case reports in regard to lipomas causing an SBO. This may have something to do with the rarity of lipomas occurring within the mesentery. However, there are case reports that, while highlighting the rarity of the condition, also mention the importance of diagnostic imaging when approaching mesenteric lipomas. One case report by Cha et al. presented a similar case where a patient developed a large mesenteric lipoma that caused intermittent abdominal symptoms such as colicky pain and constipation for years. The cause of these nonspecific symptoms went misdiagnosed as the pelvic ultrasound could not differentiate the mesenteric fat from the underlying mesenteric lipoma [10]. Another large mesenteric lipoma was reported by Vijayan et al., who also noted that while an abdominal ultrasound is the initial imaging modality of choice, it may confuse the lipomatous mass with the mesenteric fat [3]. Further imaging is needed via a contrast-enhanced computed tomography (CT) scan, which is considered the gold standard for intra-abdominal soft tissue tumors [3]. If a suspected mesenteric lipoma is not followed up with a contrast-enhanced CT scan, then the diagnosis may be missed.

After much research on mesenteric lipomas, the age of presentation seems to fluctuate. However, 44 cases of mesenteric lipoma and lipoblastoma in children under 15 years old have been reported from 1956 to 2020 [11]. One case study by Hashizume et al. researched and combined all 44 cases. Of these cases, 20 of them presented with a mesenteric lipoma, and the age of presentation for both conditions was between five months and 11 years old [11]. Also coinciding with this case, most reported mesenteric lipomas were large, averaging over 10 cm in length, and 50% were located on the mesentery of the small bowel [11]. While laparoscopic resection of mesenteric lipomas is generally well tolerated in most pediatric cases, the size of these tumors tends to be substantial [11]. The extensive size of lipomas can increase the risk of bowel obstruction, potentially leading to a surgical emergency. As demonstrated in this patient, larger mesenteric lipomas may require more invasive procedures, which in turn increases the risk of adhesions and subsequent SBO later in life. Early assessment, even when symptoms are mild, could help prevent excessive growth, minimize the need for more invasive surgical interventions, and reduce the likelihood of complications.

## Conclusions

Mesenteric lipomas are rare tumors composed of adipocytes that can present with a range of symptoms, including SBO, as seen in this case. These tumors are often difficult to diagnose due to their resemblance to normal mesenteric fat, making early and thorough imaging, particularly contrast-enhanced CT and X-rays, essential for accurate identification. Surgical resection, along with biopsy, can resolve the obstruction and provide a definitive diagnosis. While mesenteric lipomas are infrequently reported in pediatric populations, their potential to cause serious complications, such as midgut volvulus, highlights a critical gap in the literature. Further research, such as a literature review or meta-analysis of the available cases, could provide valuable insight into the presentation, management, and outcomes of mesenteric lipomas in children, helping to guide future clinical practice.
